# Continuous Positive Airway Pressure vs Mandibular Advancement Devices in the Treatment of Obstructive Sleep Apnea: An Updated Systematic Review and Meta-Analysis

**DOI:** 10.7759/cureus.21759

**Published:** 2022-01-31

**Authors:** Meghana Pattipati, Goutham Gudavalli, Matthew Zin, Lohitha Dhulipalla, Essasani Kolack, Monika Karki, Pradeep Kumar Devarakonda, Linus Yoe

**Affiliations:** 1 Internal Medicine, The Brooklyn Hospital Center, Brooklyn, USA; 2 Critical Care Medicine, Rapides Regional Medical Center, Alexandria, USA; 3 Internal Medicine, The Brooklyn Hospital Center, Brooklyn , USA

**Keywords:** osa, cpap, mad, ahi, ess, lowest oxygen saturation

## Abstract

Introduction: Obstructive sleep apnea (OSA) is the most common sleep-related breathing disorder which has various treatment options, however, continuous positive airway pressure (CPAP) remains the gold standard. The aim of this meta-analysis is to compare the current first-line treatment of OSA, i.e., the continuous positive airway pressure (CPAP) with mandibular advancement devices (MADs) in mild to severe OSA.

Objective: This meta-analysis is a comparison of the efficacy of continuous positive airway pressure vs mandibular advancement devices in patients with mild to severe obstructive sleep apnea. The primary objective of the meta-analysis is to compare the efficacy of CPAP vs MADs in the treatment of OSA. This meta-analysis includes randomized control and cross-over studies that compare the efficacy of CPAP and MAD and outcomes are reported in terms of apnea-hypopnea index (AHI), lowest oxygen saturation, and Epworth sleepiness scale both pre- and post-treatment.

Data sources and study selection: A PubMed and Cochrane database search was conducted in May 2021 and study bibliographies were reviewed. Randomized clinical trials comparing the effect of CPAP and MAD on AHI, lowest oxygen saturation, and ESS in patients with obstructive sleep apnea were selected. Of the 436 studies initially identified, eight were selected for analysis after screening. The quantitative measures used for comparing the efficacy of CPAP and MAD were post-treatment apnea-hypopnea index (AHI), lowest oxygen saturation, and post-treatment Epworth score scale (ESS).

Data extraction and synthesis: A network of meta-analyses was performed using RevMan (Copenhagen, Denmark: Nordic Cochrane Center) where multivariate random-effects models were used to generate pooled estimates. Data were analyzed using generic inverse variance method and P < 0.05 is regarded as statistically significant. Combined summary statistics of standardized (STD) paired difference in mean for individual studies and combined studies was calculated. A chi-square-based test of homogeneity was performed and the inconsistency index (I^2^) statistic was determined.

Results: Compared the AHI, lowest oxygen saturation, and ESS from baseline to follow-up pre- and post-treatment in both CPAP and MAD groups; after the database search 436 records were identified, eight studies were included in the RCT, and three were RCT crossover studies. The duration of treatment varies in each group. AHI, ESS, and lowest oxygen saturation are calculated pre- and post-treatment. Compared with MAD, CPAP was associated with decrease in AHI with a mean difference of -5.83 (95% CI, -8.85, -2.81, P < 0.01). The lowest oxygen saturation was also decreased in CPAP group compared to MAD group with a mean difference of 0.72 (95% CI, 0.51, 0.94, P < 0.01). However, there was no statistically significant difference in ESS between CPAP and MAD group with a mean difference of 0.23 (95% CI, -0.24, 0.70, P = 0.34). The meta-analysis states that among patients with obstructive sleep apnea, both CPAP and MADs are effective in reducing the AHI and lowest oxygen saturation, however, no significant difference was found in ESS pre- and post-treatment.

Conclusions: CPAP still remains the gold standard for the treatment of OSA and should continue to be recommended as a treatment for OSA. MAD can be used as adjunctive treatment or as a treatment for those who cannot readily access or do not prefer CPAP.

## Introduction and background

Obstructive sleep apnea (OSA) is the most common sleep-related breathing disorder caused by the repeated obstruction of the upper airway during sleep due to the collapse of pharyngeal muscles which leads to complete cessation (apnea) or reduction (hypopnea) of airflow and is prevalent in people with obesity. With the increase in the prevalence of obesity in the United States, the number of OSA cases will also increase. There are numerous treatment options for OSA [1]. Initial treatment of OSA can be conservative, such as weight loss via exercise programs and diet to resolve the obstruction of the airway, avoiding alcohol, smoking, and medications that relax the central nervous system. Improved sleep hygiene is also suggested to those suffering from OSA [2]. However, the current gold standard treatment is continuous positive airway pressure (CPAP), which acts by creating a positive pressure that keeps the airway open during sleep [3].

Despite CPAP’s effectiveness in treating OSA, there is low patient satisfaction and compliance with CPAP for various reasons, ranging from the cost to patient reluctance to sleep with a mask on their face at home [4]. As a result, alternatives have been searched, including mandibular advancement devices (MAD), which work by advancing the mandible during sleep and increasing the space within the airway. Mandibular advancement devices (MADs) are also often used and found to be equally effective as CPAP [3]. However, MAD is limited in its use to only mild and moderate cases of OSA and is not traditionally a replacement for CPAP in severe OSA [5]. Nevertheless, it is worth exploring the benefits of MAD in all cases of OSA compared to CPAP. This analysis aims to contribute more to already existing data on comparison of efficacy between CPAP and MAD as treatment options for OSA. This meta-analysis is an update of the meta-analysis that was performed comparing CPAP and MAD in 2017 [6]. This meta-analysis has included new studies where long-term follow-up up to 10 years was conducted [7].

Materials and methods

A database search of studies comparing the effect of CPAP and MAD was conducted in May 2021, yielding 436 total articles. In the initial search, the terms "obstructive sleep apnea," "mandibular advancement device," and "continuous positive airway pressure" were used. After screening and filtering out studies not written in English and non-comparative studies, studies not related to humans, 36 articles remained. Irrelevant articles were discarded due to lack of data, inconsistent results, and emphasis on positional OSA, leaving eight articles to be used in the analysis. These articles were of high quality based on their inclusion of multiple demographics as well as their measurement of other factors such as the BMI and lipid levels of their subjects.

The Preferred Reporting Items for Systematic Reviews and Meta-Analyses (PRISMA) criteria were used to select the studies [8]. The eligibility criteria were as follows: randomized controlled trials and randomized controlled trials (RCT) cross-over comparing CPAP to MADs in patients with mild to severe OSA who were not previously treated. The quantitative outcome measures used for the efficacy of CPAP and MAD in OSA were post-treatment apnea-hypopnea index (AHI), lowest oxygen saturation (LOS), and post-treatment Epworth score scale (ESS).

The AHI measures the severity of OSA, with a higher number suggesting more severe apnea. The oxygen saturation level is defined as the fraction of oxygen saturation in the blood, with 90% and higher being accepted as normal. The ESS is a quantitative measure of daytime sleepiness, which is a common symptom of OSA. The AHI and lowest oxygen saturation are objective measures obtained from polysomnography for diagnosis of OSA while ESS is a subjective measure self-reported by the patients. In the case of AHI and ESS, a lower number on the scale would indicate that the treatment is more effective on the apnea while in the case of lowest oxygen saturation, a higher number would indicate the treatment is more effective. To determine what data would be used and analyzed, the principal investigator ensured the articles being analyzed had similar populations in terms of age, number of patients, and duration of treatment. As the analysis is studying the effectiveness of MAD compared to CPAP, the gold standard control is CPAP and the alternative intervention is MAD.

**Figure 1:**
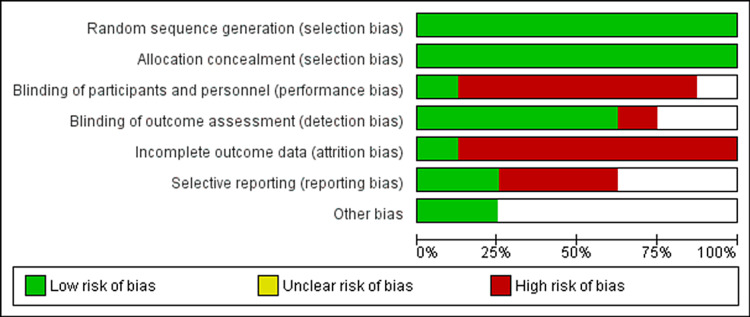
Risk of bias diagram.

The risk of bias in the meta-analysis is analyzed and inputted into Figure 1. Unfortunately, due to non-compliance of the study population, there was a high risk of attrition bias in the individual studies and therefore in the meta-analysis. Forest plots were also utilized in the analyses to calculate the risk of biases in the relevant figures. Data analysis was performed using RevMan software (Copenhagen, Denmark: Nordic Cochrane Center) wherein random effect models were used to generate pooled estimates. Data were analyzed using generic inverse variance method and P < 0.05 is regarded as statistically significant.

Combined summary statistics of the standardized (STD) paired difference in mean for the individual studies are shown. Combined STD paired differences in means were calculated and a two-sided P-value < 0.05 was considered to indicate statistical significance. A chi-square-based test of homogeneity was performed and the inconsistency index (I^2^) statistic was determined. If I^2^ was 50 or 75%, the studies were considered to be heterogeneous or highly heterogeneous, respectively. If I^2^ was 25%, the studies were considered to be homogeneous. If I^2^ statistic (> 50%) indicated that heterogeneity existed between studies, a random-effects model was calculated. Risk of bias was also tested using the funnel plot. A funnel plot is a type of scatter plot that can be useful to understand study heterogeneity of meta-analysis.

## Review

Results

**Figure 2:**
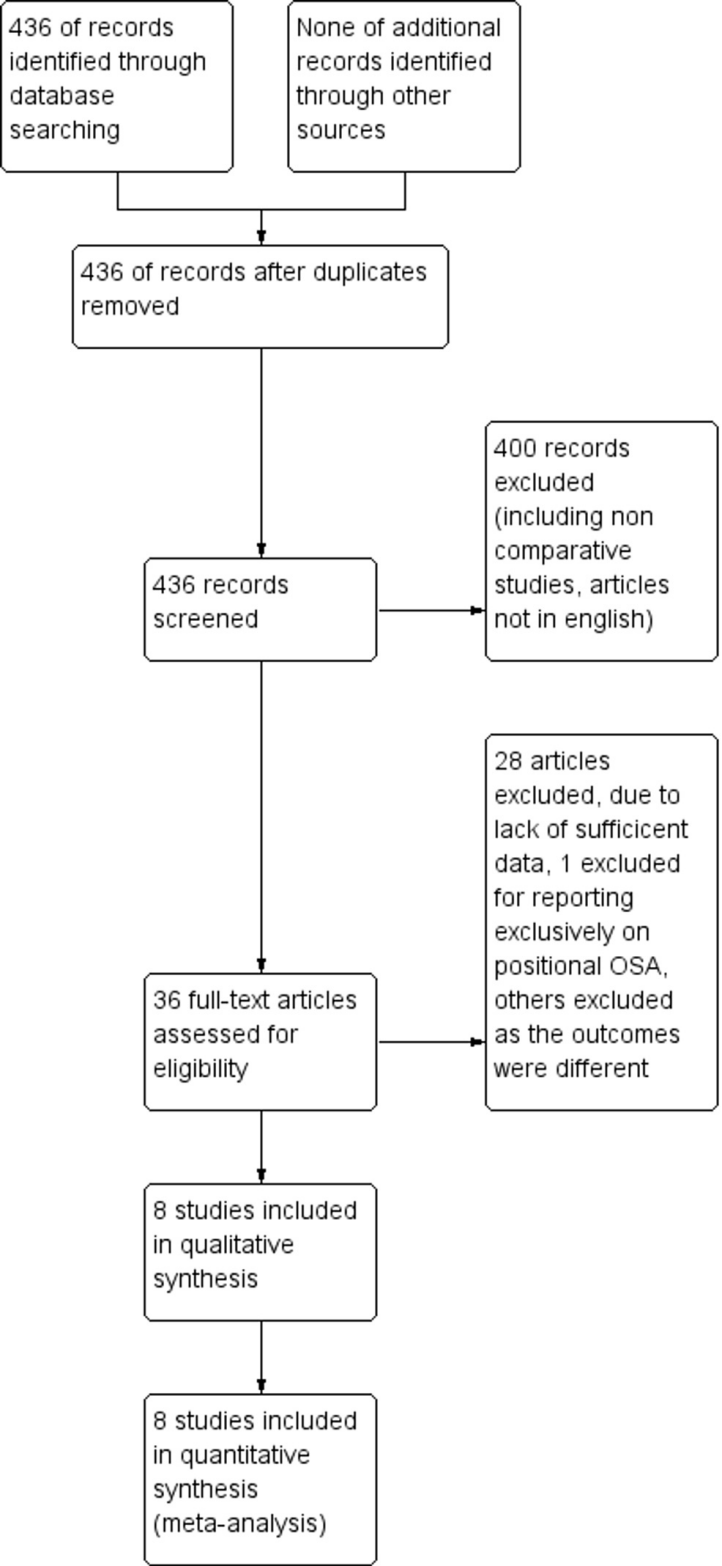
PRISMA diagram showing systematic review process. PRISMA: Preferred Reporting Items for Systematic Reviews and Meta-Analyses; OSA: obstructive sleep apnea

**Figure 3:**
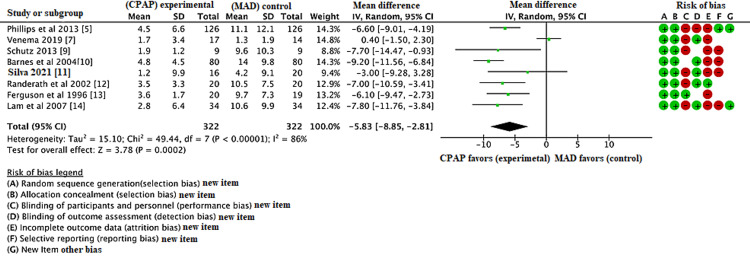
Forest plot comparing the effect of continuous positive airway pressure (CPAP) and mandibular advancement device (MAD) on the post-treatment apnea-hypopnea index (AHI).

**Figure 4:**
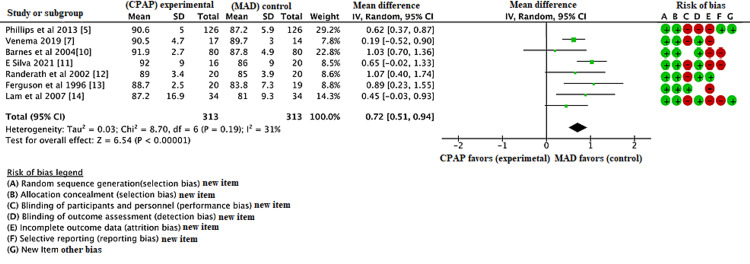
Forest plot comparing the effect of continuous positive airway pressure (CPAP) and mandibular advancement device (MAD) on the post-treatment lowest oxygen saturation level.

**Figure 5:**
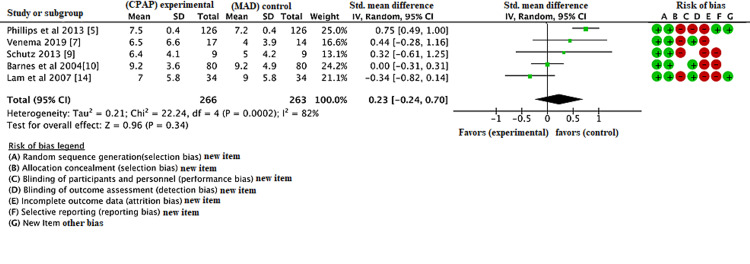
Forest plot comparing the effect of continuous positive airway pressure (CPAP) and mandibular advancement device (MAD) on the post-treatment Epworth score scale (ESS).

The database search was conducted in May 2021 and identified 436 articles. Following initial database analysis, screening, and fulfillment of eligibility criteria, eight of these articles were selected for inclusion in the study. Figure 2 represents the PRISMA flow diagram showing systematic review process. Table 1 shows data of all the included trials (body mass index {BMI}; apnea-hypopnea index {AHI}; Epworth score scale {ESS}) in the MAD group. Table 2 shows data of all the included trials (BMI; AHI; ESS) in the CPAP group. Figure 1 shows the risk of bias table above demonstrates that the areas of highest risk of bias are related to blinding, attrition, and reporting. CPAP was found to be significantly superior to MAD in reducing AHI (Figure 3). CPAP was significantly superior to MAD in raising the post-treatment lowest oxygen saturation level (Figure 4). There was no statistically significant difference found in ESS between CPAP and MAD (Figure 5).

**Table 1:** Patient demographics in MAD group. *The patients in this study participated in both treatments. BMI and AHI are the mean of all participants. **This study had patients do different duration of treatments as well. ***Study calculated the average BMI, AHI, LOS, and ESS of all participants rather than just in one type of treatment. AHI: apnea-hypopnea index; LOS: lowest oxygen saturation; ESS: Epworth score scale; RCT: randomized controlled trial; MAD: mandibular advancement device

Study	Year	Type of study	No. of patients	Duration of treatment (weeks)	BMI (mean ± SD)	Pre-treatment AHI (mean ± SD)	Pre-treatment LOS (mean ± SD)	Pre-treatment ESS (mean ± SD)
Phillips et al. [5]	2013	RCT crossover	110	4	29.5 ± 5.5	25.6 ± 12.3	82.7 ± 7.6	9.1 ± 4.2
Venema et al. [7]	2020	RCT	14	520	32.4 ± 6.6	31.7 ± 20.6	29.6 ± 6.8	10.6 ± 7.5
Schutz et al. [9]	2013	RCT	9	8	29.26 ±1.73	30.8 ± 19.0	-	6.00 ± 4.31
Barnes et al. [10]	2004	RCT	85	12	31.1 ± 0.5***	14.0 ± 1.1***	87.8 ± 0.4***	9.2 ± 0.4***
Silva et al. [11]	2021	RCT	25	26, 52**	28.2 ± 7.2	9.3 ± 5.2	84 ± 7	-
Randerath et al. [12]	2002	RCT crossover	20	6	31.2 ± 6.4	17.5 ± 7.7	83.6 ± 4.6	-
Ferguson et al. [13]	1996	RCT crossover	19*	8	30.4 ± 4.8*	24.5 ± 8.8*	83 ± 7.4	-
Lam et al. [14]	2007	RCT	34	10	27.3 ± 0.6	20.9 ± 1.7	73.8 ± 1.9	12 ± 1

**Table 2:** Patient demographics in CPAP group. *This study had patients do different duration of treatment as well. **Study calculated the average BMI, AHI, LOS, and ESS of all participants rather than just one type of treatment. ***The patients in this study participated in both treatments. BMI and AHI are the mean of all participants. AHI: apnea-hypopnea index; LOS: lowest oxygen saturation; ESS: Epworth score scale; RCT: randomized controlled trial; CPAP: continuous positive airway pressure

Study	Year	Type of study	No. of patients	Duration of treatment (weeks)	BMI (mean ± SD)	Pre-treatment AHI (mean ± SD)	Pre-treatment LOS (mean ± SD)	Pre-treatment ESS (mean ± SD)
Phillips et al. [5]	2013	RCT crossover	108	4	29.5 ± 5.5	25.6 ± 12.3	82.7 ± 7.6	9.1 ± 4.2
Venema et al. [7]	2020	RCT	17	520	33.2 ± 3.6	49.6 ± 26.1	76.7 ± 10.1	15.3 ± 3.5
Schutz et al. [9]	2013	RCT	9	8	25.90 ± 5.31	25.1 ± 10.5	-	9.88 ± 5.7
Barnes et al. [10]	2004	RCT	89	12	31.1 ± 0.5**	14.0 ±1.1 **	87.8 ± 0.4**	9.2 ± 0.4**
Silva et al. [11]	2021	RCT	31	26, 52*	28.7 ± 6.5	10.0 ± 4.6	85 ± 7	-
Randerath et al. [12]	2002	RCT crossover	20	6	31.2 ± 6.4	17.5 ± 7.7	83.6 ± 4.6	-
Ferguson et al. [13]	1996	RCT crossover	20	8	30.4 ± 4.8***	24.5 ± 8.8***	82.6 ± 6.0	-
Lam et al. [14]	2007	RCT	34	10	27.6 ± 0.6	23.8 ± 1.9	75.0 ± 1.4	12 ± 1

After the database search, 436 records were identified, eight studies included in the RCT, and three were RCT crossover studies. The duration of treatment varied in each group. BMI was included in each group. AHI, ESS, and lowest oxygen saturation were calculated pre- and post-treatment. Compared with MAD, CPAP was associated with decrease in AHI with a mean difference of -5.83 (95% CI, -8.85, -2.81, p <0.01).

The lowest oxygen saturation was also decreased in the CPAP group compared to the MAD group with a mean difference of 0.72 (95% CI, 0.51, 0.94, p<0.01). However, there was no statistically significant difference in ESS between the CPAP and MAD group with a mean difference of 0.23 (95% CI, -0.24, 0.70, p=0.34). The meta-analysis states that among patients with obstructive sleep apnea, both CPAP and MADs are effective in reducing the AHI and lowest oxygen saturation, however, no significant difference was found in ESS pre- and post-treatment.

Discussion

Increasing prevalence of obstructive sleep apnea, in addition to frequent reported patient dissatisfaction with the current gold standard therapy of CPAP, has led to the introduction of prospective alternative forms of treatment, including oral appliances such as MAD. While CPAP remains the gold standard therapy based on extensive evidence-based support, other treatments such as MAD require more investigation to support their further use. In this study, meta-analysis of the outcomes of AHI, lowest oxygen saturation level, and ESS was performed to assess the effectiveness of MAD against the gold-standard CPAP in the treatment of OSA.

The primary limitation to this study is the low number of eligible studies included for analysis [5,7,9-14]. However, seven of the eight included articles are randomized controlled trials, providing substantial evidence to the analysis. One study that is included in the meta-analysis is a recent 10-year longitudinal follow-up of patients who were initially enrolled in an RCT which yielded similar outcomes in both the CPAP and MAD groups at regular follow-up intervals up to 10 years [7]. It is worth highlighting that four of the included studies were crossover designs and duration of treatment varied between the studies. The nature of CPAP and MAD therapies, their utilization, and investigation prevent blinding of participants and investigators, thus creating a risk of bias in this category. The quantitative and subjective outcome measures of ESS, which is based on self-reported scoring by the participant, may contribute to the risk of reporting bias seen (Figure 2).

The analysis found a significant, greater decrease in AHI in patients treated with CPAP vs patients using MAD. CPAP was also associated with significantly higher lowest oxygen saturation level compared to MAD. The total ESS had no significant difference between the treatment groups. While better overall results are observed with CPAP therapy based on the outcomes investigated, patient-reported satisfaction with CPAP therapy is less convincing.

Oral appliances such as MAD have been reported to provide greater patient satisfaction, which in turn may contribute to increased patient tolerance of the therapy, higher adherence, and longer duration of treatment [15]. For these reasons, the use of oral appliances requires further evidence-based research to determine their role in the treatment of OSA. This analysis focused solely on one of the available oral appliances, MAD, while it is worth mentioning that the studies included here investigated other devices as well. This study was restricted to MAD in order to streamline the analysis.

Oral appliances such as MAD are not without side effects and potential complications, which should be mentioned when evaluating these alternative treatment options for OSA. The most common side effects of oral appliances include dry mouth, tooth or jaw discomfort, excessive salivation, and TMJ symptoms [16]. Recent studies suggest that long-term use of MAD may lead to statistically significant dental changes, and patients should therefore be counseled prior to initiating treatment [16,17]. Side effects of CPAP commonly include congestion, dry mouth, and adverse effects from the face mask itself including skin irritation and sores. Air leaks, noise complaints, and general mask discomfort are other reported side effects that may contribute to treatment failure and lack of adherence to CPAP therapy [18].

When determining treatment options for patients with obstructive sleep apnea, a thorough review of the patient’s comorbidities including maxillofacial and oral restrictions is essential. Based on the side effect profile of each therapy, CPAP vs MAD/oral appliances, therapy is likely to be more beneficial to the patient and have a higher rate of adherence if treatment is tailored and individualized. With this in mind, this analysis indicates CPAP is superior in the management of OSA when compared with MAD. MAD may be a beneficial alternative therapy in certain patients, particularly those struggling with adherence to CPAP due to the side effects mentioned above.

The findings of this analysis suggest that CPAP remains superior to MAD in the treatment of OSA. The evidence for CPAP vs MAD therapy provided here is based on a limited number of studies and further evidence-based research is required to address long-term adherence and outcomes of oral appliances such as MAD in the treatment of OSA.

## Conclusions

CPAP is the most commonly used therapy for OSA, however, the compliance with CPAP is questionable which leads our direction to use MAD as an alternative for CPAP. CPAP still remains the gold standard in the treatment of obstructive sleep apnea and should continue to be recommended as a treatment for OSA. MAD can be used as an adjunctive treatment or as a treatment for those who cannot readily access or do not prefer CPAP.
